# Artificial strain of human prions created in vitro

**DOI:** 10.1038/s41467-018-04584-z

**Published:** 2018-06-04

**Authors:** Chae Kim, Xiangzhu Xiao, Shugui Chen, Tracy Haldiman, Vitautas Smirnovas, Diane Kofskey, Miriam Warren, Krystyna Surewicz, Nicholas R. Maurer, Qingzhong Kong, Witold Surewicz, Jiri G. Safar

**Affiliations:** 1 Department of Pathology, Case Western Reserve University, 2085 Adelbert Rd, Cleveland, OH 44106 USA; 2 Departments of Physiology and Biophysics, Case Western Reserve University, 2085 Adelbert Rd, Cleveland, OH 44106 USA; 3 Department of Neurology, Case Western Reserve University, 2085 Adelbert Rd, Cleveland, OH 44106 USA; 4 Present Address: GlaxoSmithKline, 709 Swedeland Rd., King of Prussia, PA19406 UK; 5 Present Address: Department of Biothermodynamics and Drug Design, Institute of Biotechnology, Vilnius University, Graiciuno 8, Vilnius, 02241 Lithuania

## Abstract

The molecular mechanism that determines under physiological conditions transmissibility of the most common human prion disease, sporadic Creutzfeldt-Jakob disease (sCJD) is unknown. We report the synthesis of new human prion from the recombinant human prion protein expressed in bacteria in reaction seeded with sCJD MM1 prions and cofactor, ganglioside GM1. These synthetic human prions were infectious to transgenic mice expressing non-glycosylated human prion protein, causing neurologic dysfunction after 459 and 224 days in the first and second passage, respectively. The neuropathology, replication potency, and biophysical profiling suggest that a novel, particularly neurotoxic human prion strain was created. Distinct biological and structural characteristics of our synthetic human prions suggest that subtle changes in the structural organization of critical domains, some linked to posttranslational modifications of the pathogenic prion protein (PrP^Sc^), play a crucial role as a determinant of human prion infectivity, host range, and targetting of specific brain structures in mice models.

## Abstract (web summary)

Synthetic prions have previously been generated from recombinant rodent PrP. Here the authors generate synthetic human prions, by seeding human PrP with CJD prions, and characterize its infectivity in mice.

## Introduction

Human prions cause invariably fatal, rapidly progressive neurodegenerative diseases, formerly called transmissible spongiform encephalopathies (TSEs)^1,2^. Approximately 90% of human prion diseases are sporadic, 10% are associated with pathogenic mutations in PRNP gene, and 1% is caused by zoonotic or iatrogenic prion infection. Despite relatively low frequency of transmitted human prion diseases, they have gained considerable importance due to their long incubation time and high resistance of human prions to inactivation, posing unprecedented and challenging problems to disease control and public health^3^. These invariably fatal neurodegenerations are caused by a pathogenic protein, PrP^Sc^^4^, which is a misfolded isoform of the normal cellular prion protein^5–11^, PrP^C^. Human prions exist as a broad spectrum of strains corresponding to at least eight diverse clinicopathological disease phenotypes; may undergo mutation and further evolution^12^. High-resolution structure of human prions and the mechanism of their formation as well as remarkably precise anatomical targetting to different brain structures remain unknown. Many basic concepts in prion research (including that of prion strains) as well as the current strategies for therapeutic interventions were based on studies with laboratory rodent prions. Even though these studies have provided fundamentally important insights, the lessons from these studies do not apply directly to human prion diseases, as evidenced, for example, by several recently failed clinical trials^13–15^. One of the key reasons behind these disappointing results are fundamental differences between human prions causing diseases such as sporadic Creutzfeldt-Jakob disease (sCJD) and cloned laboratory prions^16^. In particular, our recent experiments indicate that replication of sCJD prions is principally determined by growth rate of prion aggregates that is in turn controled by their specific structural features, and not by their confromational stability, as it is in yeast and some murine prions^16^. How these aspects impact transmissibility of prions to man is an imperative question in light of history of iatrogenic and zoonotic transmissions and recent findings of human prions in the skin and body fluids^17,18^.

A number of recent studies have demonstrated that infectious rodent prions can be generated in vitro from bacterially expressed recombinant mouse or Syrian hamster PrP^19–23^, and the infectivity titer of some of these preparations was very high^19,21^. These studies played a fundamentally important role in providing the ultimate proof for the protein-only hypothesis of prion diseases^21,22,24–27^. However, attempts to generate infectious synthetic human prions have not yet been successful, hindering efforts to comprehensive understanding of human prion diseases such as sCJD. Here we bridge this gap, showing that human prions can be synthesized in vitro from bacterially expressed recombinant human PrP in a reaction seeded with sCJD MM1 prions in the presence of a novel cofactor, ganglioside GM1. These synthetic human prions appear to have unique strain properties. By comparing the structural organization of these synthetic human prions with that of parent sCJD MM1 prions and noninfectious human prion protein amyloid, we have identified domains in PrP that are important for the initiation of replication in vivo, i.e. their infectivity, and for targetting of different anatomical brain structures. These structural and functional insights provide a foundation for more complete understanding of the molecular basis of human prion formation and propagation.

## Results

### Generation of recombinant human prions

Using our 96-well plate-formatted quaking-induced conversion (QuIC) and the recombinant human full-length prion protein [huPrP(23–231,129 M)] substrate^16,28,29^, we first systematically screened a broad range of experimental conditions (temperature, time, recHuPrP concentration, different cofactors) for the seeded and unseeded conformational conversion reactions (Supplementary Tables 1, 2, 3, and 4). The use of conformation-dependent immunoassay (CDI) and conformational stability assay (CSA) gave us an opportunity to monitor not only the amount of the conversion product, but also to screen the conformational fidelity of the conversion reactions by comparing the conformational stability of each reaction product with that of the seed. The selection criteria for the conversion products to be used in the subsequent bioassay experiments were as follows: minimized spontaneous conversion in the absence of the seed, high yield of the conversion reaction, and similarity of conformational stability of the product and the seed. As shown in Supplementary Table 3 and Supplementary Fig. 1, in the presence of sCJD MM1 seed the reaction performed at 37 °C in a PBS (pH 6.9) containing 0.1% (w/w) Triton X100, 0.7 mM monosialoganglioside (GM1), and 0.02 mM polyadenylic acid (PolyA) was characterized both by high amplification rate as well as the final product showing conformational stability essentially identical to that of the original sCJD MM1 prion seed. Interestingly, the most efficient cofactor for amplification of sCJD MM2 prions to conformationally similar replicas was not GM1 but another lipid, 1-palmitoyl-2-oleolyl-sn-glycero-3-phospho(1’-rac-glycerol) (Supplementary Fig. 1a and Supplementary Fig. 1b, Supplementary Tables 1, 2, 3, and 4).

**Fig. 1:**
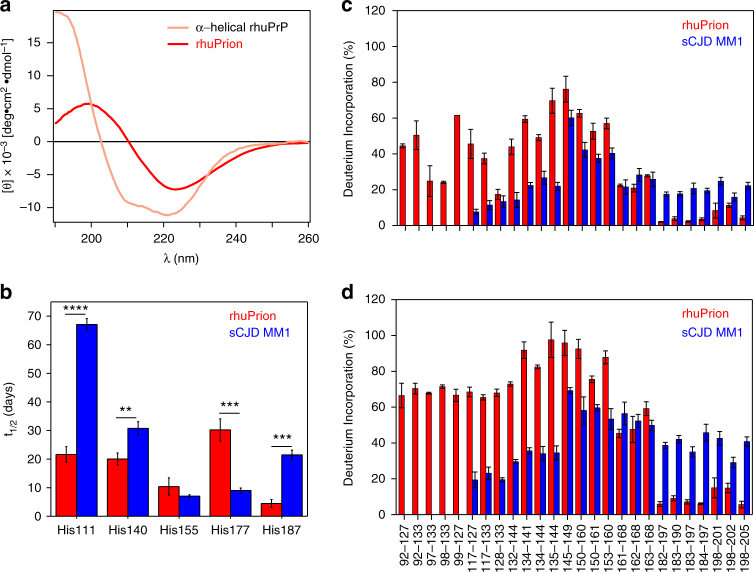
Structural comparison of rhuPrion and brain-derived sCJD MM1 PrP^Sc^ used as an initial seed. **a** Far UV circular dichroism (CD) spectrum of recombinant human PrP monomer used as a QuIC substrate, and rhuPrion. **b** Histidine H/D exchange (His-HXMS) for rhuPrion (red) and MM1 rPrP^Sc^ (blue). The parameter t_1/2_ represents the half-time of exchange reaction for individual His residues. Error bars indicate standard deviation (3 independent experiments). **p < 0.01; ***p < 0.001. **c**, **d** Backbone amide H/D exchange (HXMS) data for peptic fragments derived from rhuPrion (red) after 5 min (**c**) and 24 h (**d**) incubation in D_2_O. For comparison, previously published data are included for sCJD MM1 rPrP^Sc^ (blue) data after 5 min (**c**) and 10 days (**d**) incubation in D_2_O^16^. Error bars indicate standard deviation (3 independent experiments). *p < 0.05; **p < 0.02

Based on these data, and given that sCJD MM1 prions are known to replicate in transgenic mice much more efficiently than the MM2 conterparts^30–32^, we selected for further studies recombinant PrP replicas generated in the reaction seeded with sCJD MM1 prions in the presence of GM1. Importantly, no spontaneous (i.e., non-seeded) zconversion of recHuPrP was observed under the same experimental conditions (Supplementary Fig. 1a). First, we analyzed these replicas by far-UV circular dichroism spectroscopy (Fig. 1a), finding that they are characterized by high content of β-sheet structure (~41% vs. 3% for PrP monomer in the native conformation). Since the starting brain concentrations and purification yelds of sCJD PrP^Sc^ are very low (~200-fold lower compared to rodent PrP^Sc^), no high quality CD spectrum could be obtained for the seed. Neverthelless, the content of β-structure in the recombinant PrP sCJD replicas is similar to that previously reported for rodent prions^5^.

To further examine the degree of structural fidelity of the replication of sCJD MM1 prions using the recombinant PrP substrate and our present experimental conditions, we used two mass spectrometry-based methods: histidine hydrogen/deuterium exchange mass spectrometry (His-HXMS) and backbone amide hydrogen/deuterium exchange coupled with mass spectrometry (HXMS). These methods have been shown to be uniquely well suited for structural analysis of not only recombinant PrP aggregates but also brain-derived PrP^Sc^ ^16,33-36^.

His-HXMS monitors water accessibility of individual His side chains^37^, providing residue-specific information about the packing and interfaces between β-sheets in ordered protein aggregates^16,33,36^. As shown in Fig. 1b, four out of five His residues in the PK-resistant region are characterized by distinct environments in brain-derived sCJD MM1 PrP^Sc^ and its recombinant PrP replica, as indicated by substantial differences in the rate of H/D exchange for C2 protons in histidine imidazole rings. This clearly indicates distinct packing arrangements within the two types of PrP aggregates examined. Structural differences between sCJD MM1 PrP^Sc^ seeds and recombinant PrP products of the conversion reaction are further indicated by HXMS studies that probe the rate of H/D exchange of the backbone amide hydrogens^33–36,38^. These structural differences are particularly pronounced in the region between residues ~117 and 144. Peptic fragments from this region in the recombinant PrP product show substantially higher degree of H/D exchange compared to that in the sCJD MM1 seed already after 5 min of incubation in D_2_O (Fig. 1c), and these differences become even more dramatic after longer incubation times. Indeed, in the recombinant PrP product this region shows more than 65% exchange after 24 h, whereas in brain PrP^Sc^ it remains highly protected even after 10 days of incubation in D_2_O (Fig. 1d). By contrast, the region between residues ~182 and 205 appears to be less prone to deuterium labeling (and thus more ordered) in the recombinant PrP product than in the brain-derived sCJD MM1 seed. Altogether, these data demonstrate that, in the presence of GM1 as a cofactor, sCJD MM1 prions can efficiently seed the conversion of the recombinant PrP to β-sheet-rich aggregates. However, the structure of this product is not identical to that of the seed. One of the factors contributing to apparently imperfect conformational fidelity of this seeded conversion reaction could be the lack of post-tranlational modifications (GPI anchor, glycosylation of Asn residues) in the recombinant PrP substrate used.

### Bioassays of recombinant replicas of human prions

**Fig. 2:**
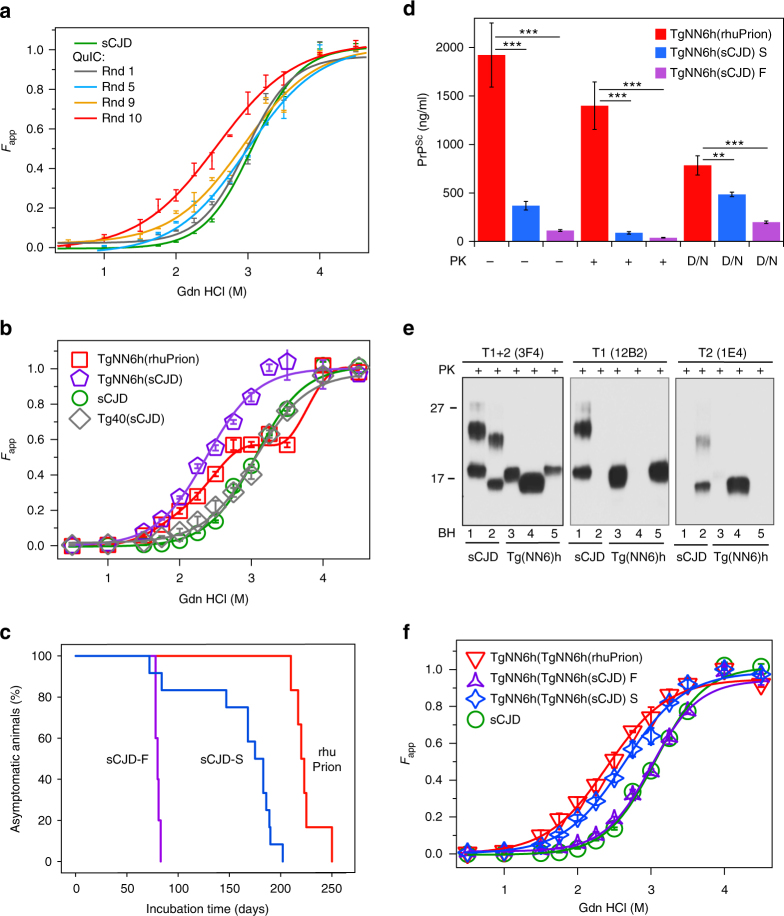
Biochemical properties of rhuPrion. **a** Conformational stability of serial QuIC products. The reaction in the first round (Rnd1) was seeded with brain-derived sCJD MM1 prions. **b** Conformational stability profiles of rhuPrion and sCJD prions after the first passage in TgNN6h or Tg40 mice. **c** Survival curves of rhuPrion, sCJD-S, and sCJD-F prions in the second passage in TgNN6h. **d** Levels of total PrP^Sc^, protease-resistant PrP^Sc^, and *D*/*N* ratio using CDI assay of brain homogenates upon the second passage of different prions in TgNN6h mice. **e** Western blots of prion isolates probed with antibodies detecting both Type 1 and Type 2 PrP^Sc^ (3F4) and antibodies specific for Type 1 (12B2) or Type 2 (1E4) PrP^Sc^: lane 1—MM1 sCJD; lane 2—MM2 sCJD; lane 3—rhuPrion; lane 4—sCJD-F prions; lane 5—sCJD-S prions. **f** Conformational stability profiles of the original human brain-derived sCJD, rhuPrion, and sCJD prions after the second passage in TgNN6h mice

To test the infectivity of the recombinant PrP replicas of sCJD MM1 prions, we performed ten rounds of QuIC (all in the presence of polyA and GM1), initially seeded with 0.1% brain homogenate of sCJD MM1 human brain. The initial reaction concentration of seed PrP^Sc^ was 0.126 ng/ml and recombinant PrP 0.1 mg/ml, corresponding to the seed/substrate mass ratio ~1:1,000,000. Each subsequent nine rounds were seeded with 10-fold diluted product from the previous round, resulting in a cumulative 10^−13^ dilution of the original brain prions and PrP^Sc^ seed to 0.1 ag of PrP^Sc^, an amount ~100,000 times below one infectious dose unit of sCJD prions^39^ (Fig. 2a). Even though the conditions were the same in all cycles, the conformational stability of the reaction products shifted gradualy in each cycle, with Gdn HCl concentration corresponding to the midpoint denaturation moving from 3.06 M for the original sCJD MM1 PrP^Sc^ to 2.56 M for the reaction product after 10 rounds of QuIC (Fig. 2a). Furthermore, less cooperative conformational transition was observed with an increase in the number of QuIC rounds. We interpret these observations as indicative of a broadening of the spectrum of conformers with lower average stability than that of the original seed.

We inoculated with the amplified material transgenic mice Tg40 that express human PrP(129 M) with native prostranslational modifications (i.e., glycosylation of Asn residues and the GPI anchor)^40^ as well as transgenic mice TgNN6h that express human PrP(129 M) in which glycosylation was abolished by N181Q and N197Q substitutions^12^. The latter line of transgenic mice was selected for high levels of cell surface expression of unglycosylated PrP^C^ after monitoring white blood cells by FACS in founder’s mice. Even though it is known that glycosylation per se has no effect on the 3D structure of PrP^41^, we further verified that the N181Q and N197Q substitutions do not affect the overall folding of the protein. Indeed, recombinant wild-type and mutant proteins show indistinguishable far-UV CD spectra and are characterized by essentially identical thermodynamic stability (Supplementary Fig. 2). It was also verified that the N181Q/N197Q PrP^C^ in TgNN6h mice is cell-surface expressed and contains the GPI anchor (as indicated by immunochemical examination of brain tissue and the release of PrP^C^ into Tx114 aqueous phase upon PIPLC treatment (Supplementary Fig. 3)^42^. While no disease was observed in Tg40 mice inoculated with the recombinant PrP replica of sCJD MM1 prions, clinical symptoms of prion disease were observed in 60% of TgNN6h mice inoculated with the same preparation, with an average incubation time of 459 days (Table 1). In control experiments it was verified that brain-derived sCJD MM1 prions (used as a seed in our conversion reaction in vitro) triggered disease in both TgNN6h and Tg40 lines, with incubation times of ~417 days and ~169 days, respectively. Neither β-sheet-rich aggregates generated without seeds according to the previously published protocol^35^, nor native monomers of recHuPrP induced clinical symptoms, and the neuropathology of aged-matched Tg40 and TgNN6h mice showed no abnormalities or deposition of pathogenic prion protein (Table 1). The conformational stability of PrP^Sc^ accumulating in Tg40 mice inoculated with sCJD MM1 prions matched the stability of the PrP^Sc^ in the inoculum. By contrast, the stability of PrP^Sc^ accumulating in the brains of TgNN6h mice inoculated with the same inoculum was significantly reduced, as indicated by lower value of [Gdn HCl]_1/2_ (Fig. 2b). TgNN6h mice inoculated with the recombinant replica of MM1 sCJD prions accumulated PrP^Sc^ characterized by biphasic denaturation curves, with ~60% of conformers having stability similar to that of PrP^Sc^ in sCJD-infected mice and ~40% having higher stability (Fig. 2b). Importantly, all TgNN6h mice inoculated with brain homogenate from the first passage of recombinant PrP prion replicas (rhuPrion) developed clinical symptoms, with the average incubation time of 224 days (Table 1; Fig. 2c). A particularly intriguing phenomenon was observed in the second passage of sCJD MM1 prions in TgNN6h mice, as these mice clearly segregated into two distinct groups: one with the average incubation time of 162 days (denoted sCJD-S, where S stands for slow) and the second one with the average incubation time of 80 days (denoted sCJD-F, where F stands for fast) (Table 1; Fig. 2c). Cumulatively, these experiments indicate that: glycosylation of the host PrP^C^ is important for conformational fidelity of sCJD prions replication in vivo, unglycosylated host PrP^C^ provides an efficient substrate for the replication of synthetic human prions (rhuPrions) generated from an unglycosylated recombinant prion protein, and sCJD prions can propagate in mice expressing unglycosylated PrP^C^ as two distinct strains, one of which is characterized by a remarkably short incubation time. Such subclonining of a single human prion isolate into two or more distinct strains has been described previously in transgenic mice^30,32^ as well as in cell cultures^43^ and led to the notion that wild prion isolates actually consist of a mixture (“cloud”) of diverse molecular assemblies^10^. Whether this strain heterogeneity observed in the present study is at least partly related to the Asn to Gln substitutions positions 181 and 197 necessary to eliminate the two N-linked glycosylation sites on human PrP is not known. However, these amino acid substitutions are generally considered as highly conservative, and circullar dichroism (CD) spectroscopy data indicate no conformational impact on PrP^C^ (Supplementary Fig. 2).

**Table 1:** Serial transmission experiments in transgenic mice expressing glycosylated and unglycosylated human PrP(129 M)

Inoculum	Host	PrP expression level	Mean IT	Prion positive	PrP^Sc^ [Gdn HCl]_1/2_
		n-fold	Days ± SEM	*n*/*n*_0_	M ± SEM
Primary host and transmission					
sCJD MM1	Human	–	–	–	3.06 ± 0.03
rHuPrion	Tg(HuPrP^N181,197Q^,129 M)	0.6	459 ± 114	6/10	2.65 ± 0.18
	Tg(HuPrP, 129 M)	1	>700	0/10	–
sCJD MM1	Tg(HuPrP^N181,197Q^,129 M)	0.6	417 ± 32	3/3	2.38 ± 0.04
	Tg(HuPrP, 129 M)	1	169 ± 12	10/10	3.07 ± 0.05
HuPrP(α-monomer)	Tg(HuPrP^N181,197Q^,129 M)	0.6	>716	0/9	–
	Tg(HuPrP, 129 M)	1	>700	0/10	–
huPrP(β-aggregates)	Tg(HuPrP^N181,197Q^,129 M)	0.6	>716	0/6	–
	Tg(HuPrP, 129 M)	1	>700	0/10	–
Secondary transmission					
rhuPrion	Tg(HuPrP^N181,197Q^,129 M)	0.6	224 ± 6	6/6	2.40 ± 0.05
sCJD MM1-F	Tg(HuPrP^N181,197Q^,129 M)	0.6	80 ± 1	5/5	3.01 ± 0.04
sCJD MM1-S	Tg(HuPrP^N181,197Q^,129 M)	0.6	162 ± 12	12/12	2.64 ± 0.04

PrP expression levels were determined relative to the expression of mouse PrP in FVB mice

*n* number of prion positive mice, *n*_0_ number of mice under observation

In the second passage of rhuPrions in TgNN6h mice, these mice accumulated ~5 and 15-fold more PrP^Sc^ than mice infected with sCJD-F and sCJD-S strains, respectively, and the same trends were observed for proteinase-resistant PrP^Sc^ (rPrP^Sc^) (Fig. 2d). Different denatured/native (D/N) ratios of PrP^Sc^ antibody epitopes (3F4 epitopes 108–112 and 8H4 epitope 175–185) indicate that they are variably exposed in the native state of mice inoculated with different inocula, confirming distinct conformations as detected by CSA (Fig. 2d). Furthermore, Western blot typing with a set of differentiating antibodies revealed that TgNN6h mice inoculated with rhuPrions accumulated Type 1 rPrP^Sc^, whereas those inoculated with sCJD-S and sCJD-F prions accumulate Type 1 and Type 2 rPrP^Sc^, respectively (Fig. 2e). While PrP^Sc^ from sCJD-F strain shows CSA profiles superimposable with those for the original human brain-derived sCJD MM1 PrP^Sc^, the nearly superimposable CSA profiles of PrP^Sc^ in rhuPrions and sCJD-S prions shifted to the lower stability (Table 1; Fig. 2f). We conclude from these observations that inoculation with rhuPrions leads to the accumulation of very high levels of PrP^Sc^ with distinct Type 1 conformational characteristics and stability, whereas the original sCJD MM1 prions cloned in TgNN6h into two isolates, one with short incubation time and Type 2 rPrP^Sc^ conformers and the other one with long incubation time and Type 1 rPrP^Sc^.

### Comparative neuropathology of rhuPrion

**Fig. 3:**
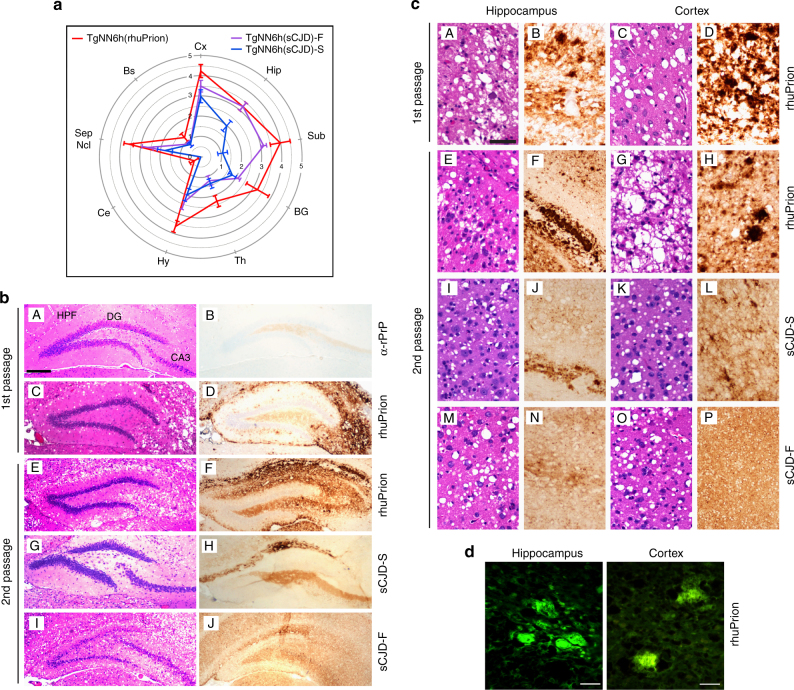
Neuropathological profiles of rhuPrion and sCJD prions in TgNN6h mice. **a** Semi quantitative vacuolization scoring within the indicated brain regions of rhuPrion and sCJD prions in TgNN6h mice: Cx, cortex; Hip, hippocampus; Sub, subiculum; BG, basal ganglia; Th, thalamus; Hy, hypothalamus; Ce, cerebellum; Sep Nlc, septal nuclei; Bs, brain stem. **b** Neuropathology panels of hippocampus formation (HPF) of age-matched TgNN6h mice inoculated with alpha-helical monomers of human prion protein (**A**, **B**), rhuPrion (**C**, **D**) in the first and (**E**, **F**) second passage; panels **G**, **H**, **I**, **J** show distinct characteristics of second passage of sCJD-S and sCJD-F prions. **c** Extensive neuronal loss and vacuolization in the CA3 pyramidal layer of hippocampus (**A**) and in the cortex (**C**) in the (first (**A**, **C**)) and second (**E**, **G**) passage of rhuPrion in contrast to less prominent loss in TgNN6h mice inoculated with sCJD-S (**I**, **K**) and sCJD-F (**M**, **O**) prions as visualized by H&E staining. Extensive PrP^Sc^ depositions in the stratum oriens of CA1 hypocampal formation and cortex following first (**B**, **D**) or second (**F**, **H**) passage of rhuPrions. Distinctly different patterns of depositions of PrP^Sc^ in sCJD-S and sCJD-F prions in both hippocampus (**J**, **N**) and cortex (**L**, **P**) of TgNN6h mice. **d** Thioflavin S positive amyloid plaques in the hippocampus and cortex of TgNN6h mice inoculated with rhuPrion. Vacuolization was visualized by H&E staining, and PrPSc deposition was assessed by immunohistochemistry with the antibody 3F4. HPF, Hippocampus formation; DG, granule cell layer of the dentate gyrus; CA3, pyramidal layer of hippocampus

Three distinct vacuolization profiles for TgNN6h mice inoculated with rhuPrion and original MM1 sCJD prions were indentified. The rhuPrion pattern exceeded the vauolization profiles of both subclones of MM1 sCJD prions, most prominently in the cortex, hippocampus, subiculum, basal ganglia, and hypothalamus. These distinct lesion profiles found in rhuPrion-inoculated TgNN6h mice were preserved upon second passage (Fig. 3a, b, c), indicating that rhuPrion represents a stable strain that is distinct from two subclones of the original sCJD MM1 prions. Remarkably, both in the first and second passage, CA3 pyramidal layer cells of hippocampus were completely destroyed and replaced by confluent vacuoles. Some of the vacuoles were proximal to large deposits of PrP^Sc^ and appeared reminiscent of florid plaques found in vCJD cases. The same hypocampal structures were either intact in sCJD-S subclone or all hypocampal formation structures were affected indiscriminately in sCJD-F subclone (Fig. 3a, b). Moreover, the first and second passage of rhuPrion led to widespread thioflavin S positive plaques of PrP^Sc^, most prominently in the hippocampus and cortex (Fig. 3c, d). In contrast, sCJD-F clone was characterized by fine, evenly distributed deposits of PrP^Sc^ with synaptic-like pattern, and the infection with sCJD-S prion clone resulted in small preamyloid deposits of PrP^Sc^ in the hippocampus and in the cortex. These data indicate that rhuPrions represent a distinct human prion strain that, apart from cortex and other gray matter, specifically targets cells in the CA3 pyramidal layer of hippocampus and leads to the accumulation of high levels of PrP^Sc^ forming amyloid plaques in the hippocampus and cortex.

**Fig. 4:**
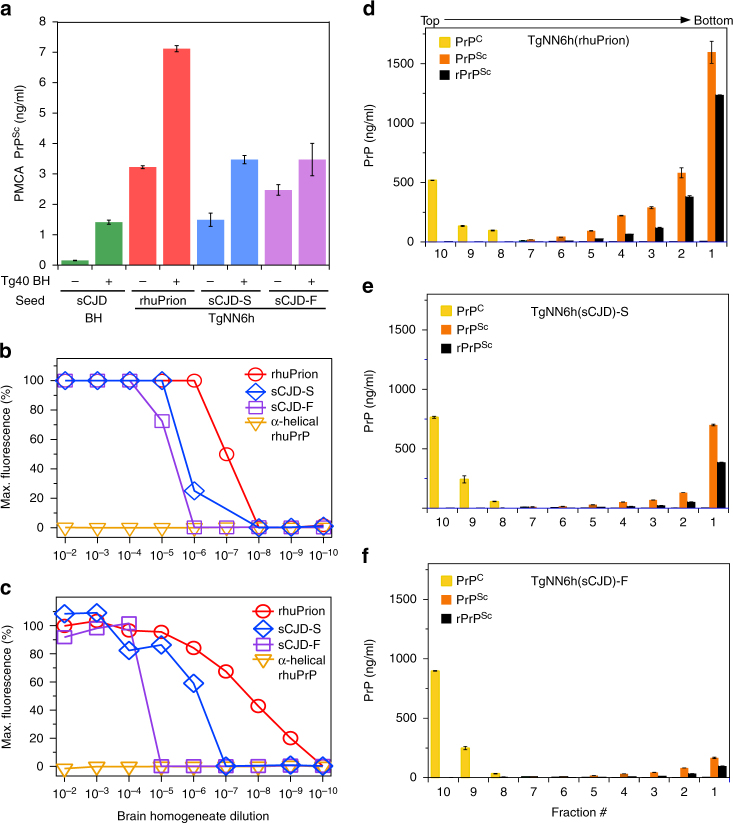
Replication potency and sedimentation velocity of rhuPrion, sCJD-S, and sCJD-F prions. **a** Amplification of rhuPrion, sCJD-S, and sCJD-F prions by sPMCA using as a substrate brain homogenate of Tg40 mice expressing fully postranslationally processed human PrP^C^ (129 M). The concentration of PrP^Sc^ before and after PMCA was measured with CDI. The second generation of RT QuIC (seeded with rhuPrion, sCJD-S, and sCJD-F prions) using recombinant SHa(PrP90-231) (**b**) and recombinant Bv(23–231) (**c**) as a substrate. Data points represent relative Thioflavin T fluorescence of the positive control reference and end point dilution of the seeds in four RT QuIC experiments. Sedimentation velocity profiles of rhuPrion (**d**), sCJD-S (**e**), and sCJD-F prions (**f**). The samples were fractionated by ultracentrifugation in sucrose gradient, and fractions were collected from the bottom of the tubes and analyzed for PrP^Sc^ by CDI. The bars represent average ± SEM; CDI was performed on each sCJD sample in triplicate

Second passage rhuPrions from TgNN6h mice brains could be replicated in vitro by protein misfolding cyclic amplification using Tg40 mice brain homogenate as a substrate. The replication rate was paradoxically lower for sCJD-F subclone, and both rhuPrion and sCJD-S replicated with comparable rates (Fig. 4a). These results indicate that unglycosylated prions present in TgNN6h isolates, including rhuPrions, can be replicated using fully posttranslationally processed normal human PrP^C^ substrate. The end-point titration of seeding activity of all three TgNN6h prion isolates in RT QuIC with SHa(PrP90-231) (Fig. 4b) and with Bv(23–231) (Fig. 4c) substrates indicates that rhuPrion has a seeding titer ~100-fold and 1000-fold higher compared to sCJD-S and sCJD-F prions, respectively. Since the concentration of PrP^Sc^ in rhuPrions brain homogenates was only ~twofold higher than that of PrP^Sc^ in sCJD-S and sCJD-F homogenates (Fig. 4a), this remarkably high seeding potency suggest that rhuPrion is composed of unique conformers with exceptionally high capacity to convert PrP monomers into Thioflavin-positive β-sheet aggregates. The nonequlibrium sedimentation velocity profile of rhuPrion and both sCJD isolates were simillar, with the highest concentration of PrP^Sc^ in the botom of the tubes (Fig. 4d, e, f). Based on previous calibration^29^, we estimate that the prevalevant population of aggregates in rhuPrions has a mass of at least 14 × 10^6^ Da, which corresponds to at least 600 monomers of PrP^Sc^.

## Discussion

The pathogenic conformer of human prion protein—PrP^Sc^—is an essential and possibly the only constituent of infectious human prions.. However, a high-resolution structure of human PrP^Sc^ is lacking, and the molecular mechanism that determine initiation and propagation under physiological conditions—prion infectivity—remain obscure^16,44^. Our present study demonstrates, for the first time, that infectious human prion can be generated in vitro from the bactrially expressed recombinant human prion protein in a reaction seeded with brain-derived sCJD prions. Furthermore, our experiments reveal that, even though many conditions are conductive to efficient propagation of PrP aggrergates in vitro upon seeding with human prions, they do not always cause the development of prion disease in vivo. The infectivity and pathogeneicity, as well as specific targetting of neurons in different brain stuctures in vivo apear to be controled by by seemingly subtle differences in the conformation, especially in the C-terminal domain that of PrP that is involved in posttranslational modifications.

Although the infectivity of the herein described new artificial prion in the first passage was relatively low and limitted to the transgenic mice line expressing unglycosylated human PrP, the bioassay revealed a particularly destructive neuropathological phenotype with widspread vacuolization and loss of neurons, particularly in the CA3 (Cornu Ammonis) region of hippocampus, and numerous Thioflavin S-positive plaques. The CA3 region has attracted major attention in recent years for its specific role in memory processes and in susceptibility to seizures, and the synthetic human prion offers a new powerful tool to study the pathophysiology of neurodegeneration in this area, especially relevant to Alzheimer’s disease. While these intriguing findings may suggest generally lower fidelity of in vitro replication of brain derived prions with unglycosylated substrate, another possibility is that posttanslational modification of prion protein—glycosylation and glycosylphosphatidylinositol (GPI) anchor—are important determinants of conformational fidelity in serial propagation in vitro and also in the transmissibility (infectibility) of prions in vivo^45^. Even though some aspects of the latter possibility have recently been suggested based on experiments with cell-adapted prions and rodent prion strains^43,46,47^, to fully understand the impact of postranslational modifications on the structural fidelity of human prion replication to the infectious form, it will be critical to perform a structural and functional comparison of conversion reactions using recombinant PrP substrates in which one can systematically alter the type of specific modifications.

The discovery of variable glycosylation patterns and heritable polymorphic PK cleavage sites of human brain PrP^Sc^ markedly differentiated early on human prions from cloned laboratory prions of animal origin^48–50^. The subsequent experiments with more sensitive biophysical tools revealed a wide spectrum of sCJD PrP^Sc^ structural states and interplay with distinct glycosylation patterns in each clinicopathological category of human prion diseases, and argued for a broad spectrum of human prion strains coding for different phenotypes^29,51,52^. Moreover, our most recent data demonstrate that typical human prions causing sporadic Creutzfeldt-Jakob disease (sCJD) differ in a major way from both cloned laboratory prions and synthetic prion amyloids^16^. Importantly, phenotypically very distant MM1 and MM2 sCJD prions differ in their structural organization, specifically in the polypeptide beckbone and quaternary structure (packing) of prion particles. These unique structural features primarilly control the replication rate of human prions^16^. Finaly, our in vitro data reported here suggest that this aspect is in turn controlled (probably kinetically) by strain-specific auxiliary molecules differentially facilitating conversion reactions of some prion strains but not others.

The existence of auxiliary molecules facilitating PrP^Sc^ formation and replication was first suggested from the co-purification of Syrian hamster PrP^Sc^ with sphingolipids, galactosylceramide, and sphingomyelin^53^, and with 25-mer polynucleotides encoding variable sequences^39^. This hypothesis was supported more recently by the findings that the PMCA conversion of purified brain-derived PrP^C^ to infectious conformers could be accomplished only in the presence of polyanions such as RNA or in a case of recombinant PrP with RNA and synthetic lipids^19,54^. As a starting point for our sCJD prions (MM1 and MM2)-seeded conversion reactions, we used QuIC in the presence of polyA, Monosialoganglioside GM1 from bovine brain (GM1), or synthetic 2-Oleoyl-1-palmitoyl-sn-glycero-3-phospho-rac-(1-glycerol) (POPG). While the replication and conformational fidelity of MM1 sCJD prions was facilitated by GM1, MM2 sCJD was facilitated by POPG. Considering structural and charge heterogeneity of gangliosides, and their localization together with PrP^C^ in cholesterol rich domains (caveoli), their distinct characteristics and levels in different cells may explain preferential cellular targetting of different human prions and thus distinct neuropathological profiles. These results support a critical role of cofactors in the strain-specific formation and propagation of human prions, and suggest that different cofactors guide human PrP misfolding in distinct pathways, resulting in different prions. However, whether additional cofactors can also facilitate strain-specific replication of human prions is at present unknown and these initial observations have to be extended with screenining of additional cofactors. The obvious potential candidates are phosphatidylglycerol or phosphatidylethanolamine alone, as these cofactors have been shown to facilitate PMCA conversion of bacterially expressed mouse PrP to highly infectious prions^26,54,55^. Additional human prion strain-specific cofactors can be potentially identified by analysis of nonproteinaceous molecules associated with purified human prions employing a strategy similar to that used previously for identification of rodent prion cofactors^56–58^. These investigations could open new therapeutic strategies for human prion disease by designing synthetic analogs of auxiliary factors with inhibitory or blocking effect on human prion replication.

## Methods

### Ethics statement

All procedures were performed under protocols approved by the Institutional Review Board at Case Western Reserve University. In all cases, written informed consent for research was obtained from the patient or legal guardian and the material used had appropriate ethical approval for use in this project. All patient data and samples were coded and handled according to NIH guidelines to protect patient identities. All animal studies were conducted under the protocols approved by Case Western Reserve University Institutional Animal Care and Use Committee (IACUC).

### Patients and clinical evaluations

Prion disease cases were reviewed pertaining to updated diagnostic criteria for sCJD^59^. Brain MRIs were considered positive for CJD if the reading by experienced radiologist fulfilled the criteria defined peviously^27^.

The criteria for inclusion were availability of a clinical diagnosis of CJD according to WHO criteria^59–62^ with dated initial symptoms to ascertain the disease duration; methionine or valine at codon 129 of the human prion protein (PrP) gene (*PRNP*); unequivocal classification of Type 1, Type 2, or Type 1–2 rPrPSc sCJD according to Western blot (WB) pattern; and unequivocal classification of pathology as definite Type 1, Type 2, or mixed Type 1–2^63,64^. The criteria for diagnosis of rapidly progressive Alzheimer disease (AD)^63^ were as follows: probable clinical diagnosis of AD^63,65^; absent autosomal dominant pattern of dementia; unequivocal classification as AD after detailed neuropathology and immunohistochemistry of tau proteins and amyloid beta using the current National Institute of Aging—Alzheimer’s Association guidelines^63,66^.

### CJD Classification, brain samples, and *PRNP* gene sequencing

DNA was extracted from frozen brain tissues in all cases, and genotypic analysis of the *PRNP* coding region was performed as described^39,67,68^. The CJD cases were classified on the basis of diagnostic pathology, immunohistochemistry, and WB examination of 2 or 3 brain regions (including frontal, occipital and cerebellum cortices) with mAb 3F4^3,12,29,52^.

For molecular analyses, we used 200–350 mg cuts of frontal (superior and more posterior middle gyri) brain cortex stored at −80 °C after autopsy. The other cerebral hemisphere was fixed in formalin and 16 anatomical areas were used for histopathological and immunohistochemical classification of prion disease according NPDPSC’s standard protocols^3,23,69^. In a case of equivocal classification of sCJD subtype between pathology and Western blots, we based the classification on the molecular characteristics of PrP^Sc^ on Western blots developed with a panel of Type 1 and Type 2-specific antibodies as described previously^3,70,71^.

### Bioassays in transgenic mice expressing human PrP^C^ (129M)

The human PrP^N181Q/N197Q^ ORF, which contains 2 point mutations that change Asn to Gln in amino acid positions 181 and 197, thereby eliminating the two N-linked glycosylation sites on human PrP, was generated by PCR mutagenesis from the human PrP-129M transgene construct used for the generation of the Tg40 mice and described previously^40^. The homozygous TgNN6h line expressing the human PrP^N181Q/N197Q^ ORF at about 60% of the PrP level found in wild type FVB mice was described previously^12^. To assay for prion infectivity in the TgNN6h mice, 30 µl of the sample (brain homogenates or recombinanat human PrP preparations before or after QuIC) in PBS was injected into the mouse brain with a 26 gauge needle inserted to a depth of about 4 mm at the left parietal region of the cranium and the symptoms monitored as described previously^40^. To prevent cross contamination, the intracerebral inoculations of synthetic prions were performed under sterile conditions in days when no other inoculations where carried out. The animals were housed in microisolator cages and stringent aseptic and biosafety techniques were followed for animal handling and husbandry as we described previously^40^. Within three days after the appearance of symptoms or at death, the animal was euthanized and the brain was removed; one-half was frozen for biochemical studies, and the other half was stored in formalin for histology and immunohistochemistry analysis as described below.

### The sCJD brain homogenates

We selected brain samples from a group of sCJD patients, all of whom were methionine or valine homozygous at codon 129 of the human prion protein (PrP) gene (PRNP), and who were classified according to pathology and WB pattern as pure Type 1 or Type 2 sCJD at the National Prion Disease Pathology Surveillance Center (NPDPSC) in Cleveland, OH. DNA was extracted from frozen brain tissues in all cases, and genotypic analysis of the PRNP coding region was performed as described^67,68^. All patient data and samples were handled with IRB approval according NIH guidelines to protect patients’ identities and the patients or their legal representatives signed written consent.

Slices of human brain tissues weighing between 200 and 350 mg from the frontal cortex were first homogenized to a final 15% (w/v) in calcium- and magnesium-free PBS, pH 7.4, by using Mini-Beadbeater 16 Cell Disrupter (Biospec, Bartlesville, OK). The homogenates were then diluted to a final 10% (w/v) in PBS / 1% Triton X-100 / 5 mM EDTA / 1× Complete PI cocktail (Roche Applied Science, Indianapolis, IN), pH 7.4. They were clarified by a centrifugation at 500×*g* for 5 min, and the collected supernatant was aliquoted and stored at −80 °C for QuIC.

### QuIC

The QuIC was performed as described^72^ with following modifications. The recombinant human PrP(23–231) with codon 129 M or 129 V used as a substrate in QuIC were expressed, purified, and refolded to α-helical conformation as described previously^73^. The initial concentration of recombinant human PrP(23–231) was calculated from absorbance at 280 nm and molar extinction coefficient 56,650 M^−1^ cm^−1^. The stock of rechuPrP(23–231) in 10 mM Sodium Acetate buffer, pH 4.0, was pretreated with 12 mM HCl at 1:5.0 (rechuHuPrP: HCl, v/v) ratio for 7.5 min and immediately diluted to final 0.1 mg/ml into the basic reaction buffer composed of 20 mM NaH_2_PO_4_, 130 mM NaCl, pH 6.9. The recHuPrP concentration, QuIC time, and composition of alternative buffers containing 0.1% (v/v) Triton X-100, 1:5000 (v/v) N2 Supplement (Invitrogen, Carlsbad, CA), 0.1% (w/v) SDS, polyadenylic acid (Poly A, Sigma, St. Louis, MO), Monosialoganglioside G_M1_ from bovine brain (G_M1_, Avanti Polar Lipids, Alabaster, AL), or synthetic 2-Oleoyl-1-palmitoyl-sn-glycero-3-phospho-rac-(1-glycerol) (POPG, Sigma, St. Louis, MO) are described in the Supplementary Tables 1, 2, 3, and 4. The QuIC was performed with final volume 100 µl per well in sterile V-bottom, low-binding polypropylene 96-well plate (VWR, Arlington Heights, IL) equipped with a 3 mm diameter PTFE bead (Fisher Scientific, Pittsburg, PA) in each well. The aliquots of sCJD brain homogenates used as seeds were sonicated 3 × 5 s at 60% power setting using Misonix Sonicator S-4000 (Qsonica, Newtown, CT), diluted immediately into the complete QuIC reaction buffer to obtain final 10^−4^ dilution of sCJD brain prions, and the plates were sealed with sterile Axymat* Silicone Sealing Mat (VWR, Arlington Heights, IL). The QuIC was performed at 37, 45, or 55 °C for 5 to 96 h in Eppendorf Thermomixer (Eppendorf, Hauppauge, NY) set for 1 min shaking at 1400 rpm followed by 1 min incubation. The control unseeded samples of spontaneously converted prion protein amyloid were prepared according previously published protocol^35^.

### PK treatment

To each well containing 100 µl of QuIC reaction buffer was added 50 µl of PBS, pH 6.9, containing 3% (w/v) Sarcosyl and Proteinase K (PK, Amresco, Solon, OH) to obtain the final Sarcosyl concentration 1% (w/v) and PrP/enzyme ratio 10:1 (w/w) or 100:1 (w/w). The plates was incubated for 1 h at 37 °C at 1200 rpm on the Eppendorf Thermomixer with 1-min. interval. Alternatively, QuIC reaction performed in PBS, pH 6.9 containing 0.5 M NaCl and no detergents was followed with PK treatment without detergents. The PK was blocked in each well with protease inhibitors (0.5 mM PMSF final, and 5 ug/ml of aprotinin and leupeptin).

### SDS PAGE and western blots

The samples were mixed 1:1 with WB (Laemmli Buffer (Bio-Rad, Hercules, CA) containing 5% (v/v) beta-mercaptoethanol (BME), 2% (v/v) SDS, and 115 mM Tris-HCl, pH 6.8 and heated at 100 °C for 5 min. Either 150 ng (silver staining) or 2 ng (Western blots) per lane were loaded on 1 mm 18% Polyacrylamide Tris-HCl, SDS-PAGE gels (Bio-Rad).

SDS-PAGE gels were transblotted to Immobilon-P Transfer Membranes (Millipore, Bedford, MA) in Bio-Rad Western Blot apparatus. The filters were blocked with 5% (w/v) Nonfat milk in TBS containing 0.1% of Tween 20 (v/v) (Sigma, St. Louis, MO), and incubated with one of the following antibodies: mAb 3F4^74^ (epitope residue 107–112); mAb 12B2 (epitope residues 89–93, kindly provided by Jan Langeveld^75^, and mAb 1E4 (epitope residues 97–108, Cell Sciences, Canton, MA). After incubation with secondary anti-mouse IgG peroxidase-labeled sheep Ab (Amersham, Piscataway, NJ), the membranes were developed with the ECL Plus detection system (Amersham) and exposed to Kodak BioMax MR Films (Fisher Scientific) or Kodak BioMax XAR Films (Fisher Scientific).

### Histology and immunohistochemistry lesion profiles

We used hematoxylin and eosin or antibody 3F4 on four different coronal sections for the histopathology and immunohistochemical detection of PrP^Sc^ in rodent brains as reported previously^39,76,77^. Semiquantitative evaluation of spongiosis and gliosis was performed by comparing hematoxylin and eosin-stained sections using a modified procedure of Fraser and Dickinson^78^. Nine areas of gray matter were examined, and a score was given for the severity of spongiform degeneration using the scale based on a number and confluency of vacuoles in the field at 400-fold magnification: 0, no vacuoles; 1, one to three vacuoles widely and unevenly distributed; 2, on average ten vacuoles evenly scattered; 3, on average thirty vacuoles evenly scattered; 4, on average 90 vacuoles with some confluences; 5, dense vacuolation with proiminent confluence.

### Thioflavin S staining

After deparaffinization, formalin-fixed sections were stained in Thioflavin S for 7 min, washed three times in 80% alcohol, dehydrated in ethanol, cleared in xylene, and cover slipped with Vectashield mounting medium for fluorescence. Sections were kept in the dark at 4 ˚C for 30 min before being viewed under the fluorescence microscope (Olympus IX71).

### Conformation-dependent immunoassay (CDI)

The CDI for human PrP was performed as described previously^39,79^, with following modifications: white Lumitrac 600 High Binding Plates (E&K Scientific, Santa Clara, CA) were coated with mAb 8H4 (epitope 175–185)^80^ and aliquots of each sample containing 0.007% (v/v) of Patent Blue V (Sigma) were dilutedinto plate wells filled with 200 µl of Assay Buffer (Perkin Elmer, Waltham, MA); he captured PrP was detected by a europium-conjugated^81^ anti-PrP mAb 3F4 (epitope 108–112)^74^ and the time-resolved fluorescence (TRF) signals were measured by the multi-mode microplate reader PHERAstar Plus (BMG LabTech, Durham, NC).

### Conformational stability assay (CSA) of prions

The sequential denaturation of human PrP^Sc^ was performed as described previously^81^, with several modifications. Frozen aliquots of PrP^Sc^ were thawed, sonicated 3 × 5 s at 60% power with Sonicator 4000 (Qsonica, Newtown, CT); and the concentration was adjusted to constant ~50 ng/ml of PrP^Sc^ or rhuPrion. The 15 µl aliquots in 15 tubes were treated with increasing concentrations of 8 M GdnHCl containing 0.007% (v/v) Patent Blue V (Sigma, St. Louis, MO) in 0.25 M or 0.5 M increments. After 30 min incubation at room temperature, individual tubes were rapidly diluted with Assay Buffer (Perkin Elmer, Waltham, MA) containing diminishing concentrations of 8 M GdnHCl, so that the final concentration in all samples was 0.411 M. Each individual aliquot was immediately loaded in triplicates to dry white Lumitrac 600, High Binding Plates (E&K Scientific, Santa Clara, CA), coated with mAb 8H4, and developed in accordance with CDI protocol using europium-labeled mAb 3F4 for detection^39,81–83^. The raw TRF signal was converted into the apparent fractional change of unfolding (Fapp) as follows: Fapp = (TRF_OBS_- TRF_N_)/(TRF_U_-TRF_N_) where TRF_OBS_ is the observed TRF value, and TRF_N_ and TRF_U_ are the TRF values for native and unfolded forms, respectively, at the given Gdn HCl concentration^5^. To determine the concentration of Gdn HCl where 50% of PrP^Sc^ is unfolded ([Gdn HCl]_1/2_), the data were fitted by least square method with a sigmoidal transition model as described previously^52^.

### Second-generation RT-QuIC

The second-generation RT-QuIC assays were performed as previously described, with minor modifications^18^. The RT-QuIC reaction mix was prepared as follows: 10 mM phosphate buffer (pH 7.4), 300 mM NaCl, 0.1 mg/ml SHarPrP(23–231), 10 µM thioflavin T (ThT), 1 mM ethylenediaminetetraacteic acid tetrasodium salt (EDTA) and 0.002% sodium dodecyl sulfate (SDS). The 85 µl of reaction mix was loaded into each plate well and seeded with 15 µl of brain homogenates for a final reaction volume of 100 µl. The incubation and real time fluorescence monitoring was performed using the FLUOstar Omega plate reader (BMG LABTECH GmbH, Ortenberg, Germany) set to the following parameters: 55 °C incubation, 60 h reaction time, 60 s shake/60 s rest cycles with ThT fluorescence measurements taken every 45 min.

### HXMS experiments

The pellets containing aggregates of rechuPrP after PK treatment were washed three times in H_2_O. For deuterium labeling, pellets (~20 µl) were resuspended in 400 μl of the buffer (10 mM phosphate, pH 7.0) prepared in D_2_O. After incubation at room temperature for different time periods, samples were cetrifuged at 21,000×*g* for 5 min and dissociated by incubating for 5 min on ice in a quenching buffer (0.1 M phosphate, pH 2.4) containing 7 M guanidine hydrochlorode and 0.1 M TCEP [Tris(2-carboxyethyl)phosphine hydrochloride]. The samples were then diluted 10 times with an ice cold 0.05% TFA (Trifluoroacetic acid) and digested for 5 min with agarose-immobilized pepsin (Thermo Scientific). The peptic fragments were separated and analyzed by mass spectrometry as described previously^34,35^.

### His-HXMS experiments

For these measurements, the pellets containing aggregates of rhuPrP after PK treatment (~3.6ug) were suspended in D2O buffer (10 mM sodium phosphate, pH 9.0) and incubated for 5 days at 37 °C. Following incubation,samples were collected by centrifugation. To obtain peptide fragments containing single His residues, the aggregates were dissociated with 7 M Gdn and digested first with agarose-immobilized pepsin and then silica-immobilized trypsin as descibed previously^16^. The peptic fragments were separated on an UPLC column and analyzed by mass spectrometry on an LTQ Orbitrap mass spectrometer. Mass spectrometric data were analyzed and half-life (*t*_1/2_, days) of His exchange reaction was determined as described previously^16^.

### Statistical analysis

We compared the parameters for the whole group and different phenotypic subgroups with ANOVA or Fisher exact test. Cumulative survival curves were constructed by the Kaplan–Meier method and compared using the Mantel-Cox and Breslow methods. For each type of PrP^Sc^ and *PRNP* codon 129 polymorphism, we report descriptive statistics and each variable was compared with ANOVA.

### Data availability

All data supporting the findings in this study are available within the article and Supporting files, or are available from the corresponding author upon reasonable request.

## Electronic supplementary material

Supplementary Information

Peer Review File
