# Supplementary material for: Artificial strain of human prions created in vitro
Source: Nat Commun. 2018 Jun 4;9:2166. doi: 10.1038/s41467-018-04584-z (PMC5986862; doi:10.1038/s41467-018-04584-z)
Supplement: Supplementary file 1 — Supplementary Information [file 41467_2018_4584_MOESM1_ESM.pdf]

ARTIFICIAL STRAIN OF HUMAN PRIONS CREATED IN VITRO

Supplementary Information

Correspondence to
Jiri G. Safar
jgs109@case.edu

SUPPLEMENTARY FIGURES AND TABLES

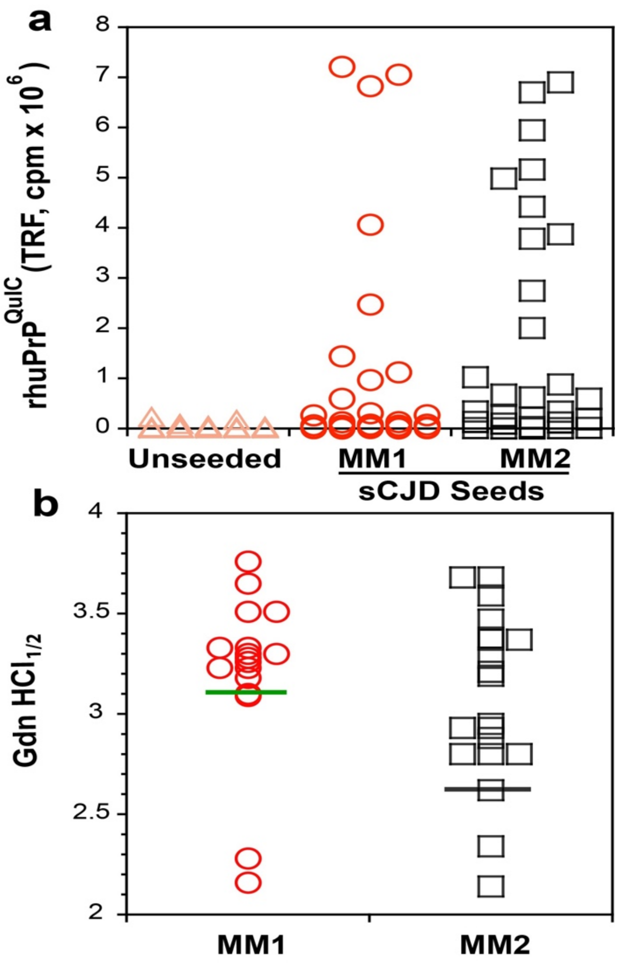

Supplementary Figure S1. Each data point represents different QuIC reaction performed unseeded or seeded with MM1 and MM2 sCJD prions. The (Fig. S1a) level and (Fig. S1b) conformational stability of PK-resistant reaction product was obtained for particular condition from two independent QuIC reactions and measured in triplicate after Protease K treatment with CDI and CSA. The green and black lines in Fig. S1b indicate conformational stability of seed MM1 and MM2 prions, respectively. Detail reaction condition, cofactor, and output data for each experiment are listed in Supplemental Tables 1, 2, 3, and 4. The conformational stability of the product was determined by CSA and Gdn HCl$_{1/2}$ values represent Gdn HCl concentration corresponding to the midpoint of the transition from native to denatured state of PrP

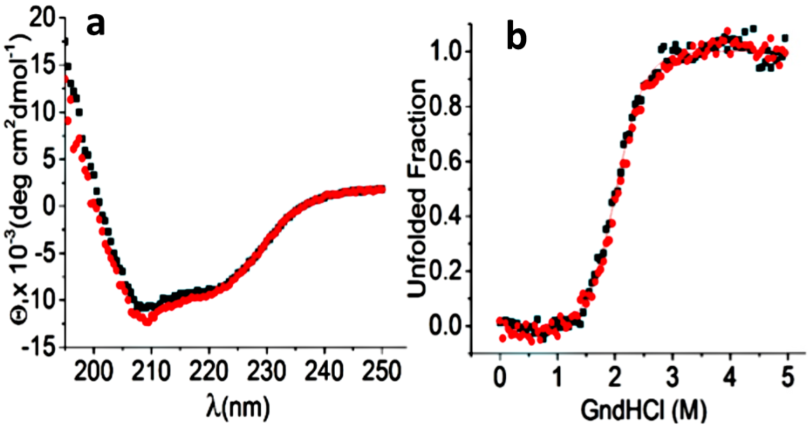

Supplementary Figure S 2. N181Q and N197Q substitutions do not affect the overall folding of PrP. (a) Far UV circular dichroism spectra of wild-type (black) and N181Q/N197Q (red) recombinant PrP. (b) Stability curves for wild-type (black) and N181Q/N197Q (red) recombinant PrP as assessed by equilibrium unfolding in GdnHCl. The unfolding reaction was monitored by ellipticity at 222 nm.

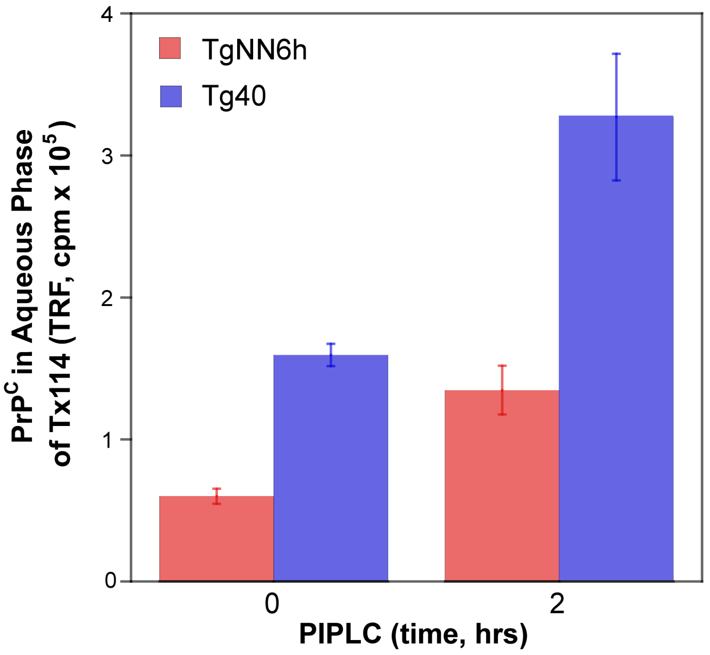

Supplementary Figure S3. Cleavage of the glycolipid of PrP^C in TgNN6h (red) and Tg40 (blue) brain homogenates with Phosphatidylinositol-Specific Phospholipase C (PIPLC) and monitored by Triton X114 partitioning [83]. The PrP^C release into the aqueous phase was measured by CDI in triplicate.

Supplementary Table 1. Reagents used in QuIC experiments reported in Supplemental Tables 2, 3, and 4.

Substrate	Reaction Buffer	Additive	Final Concentration
recHuPrP(23-231,129M)	PBS, pH 6.9	NaCl	500 mM
	NaCl 130mM	Triton X (Tx)	0.1% (w/v)
	NaH_2PO4 20mM	Sodium dodecyl sulfate (SDS)	0.1% (w/v)
		N-2 Supplement (N2)	1:5000 (v/v)
		Monosialoganglioside GM_1 (GM1)	0.694 mM
		Polyadenylic acid (Poly A)	0.016 mM
		2-Oleoyl-1-palmitoyl-sn-glycero-3-phospho-rac-1-glycerol (POPG)	0.122 mM

Supplementary Table 2. Unseeded QuIC. The average levels and conformational stability of PK-resistant reaction products were obtained from two independent QuIC reactions measured in triplicate with CDI. The CDI signal in TRF (cpm) is proportional to the concentration of PK-resistant PrP.

Seed	Additive	recHuPrP	QuIC Time	PK-resPrP		
		μM	hrs	CDI TRF, cpm ± S.E.M.		
Blank	GM1	4.2	48	1773	±	450
Blank	GM1	4.2	96	7986	±	459
Blank	NaCl	1.0	24	3715	±	1300
Blank	NaCl	2.1	24	7426	±	1059
Blank	NaCl/GM1	4.2	48	16431	±	1538
Blank	SDS/Tx/N2	4.2	24	25499	±	248
Blank	SDS/Tx/N2	4.2	48	34792	±	709
Blank	SDS/Tx/NaCl	4.2	24	51972	±	9023
Blank	SDS/Tx/NaCl	4.2	48	89678	±	4225
Blank	Tx/GM1	4.2	24	18250	±	5702
Blank	Tx/GM1	4.2	48	16581	±	3389
Blank	Tx/Poly A/GM1	1.0	24	2380	±	61
Blank	Tx/Poly A/GM1	1.0	48	2846	±	910
Blank	Tx/Poly A/GM1	2.1	24	4957	±	1458
Blank	Tx/Poly A/GM1	2.1	48	9088	±	2993
Blank	Tx/Poly A/GM1	4.2	48	6933	±	674
Blank	Tx/Poly A/GM1/NaCl	4.2	48	9984	±	1961
Blank	Tx/POPG/GM1	4.2	24	27771	±	7193
Blank	Tx/POPG/GM1/NaCl	4.2	48	23231	±	779
Blank	Tx/POPG/Poly A	1.0	24	2051	±	57
Blank	Tx/POPG/Poly A	1.0	48	2674	±	692
Blank	Tx/POPG/Poly A	2.1	24	1361	±	45
Blank	Tx/POPG/Poly A	2.1	48	2273	±	651
Blank	Tx/POPG/Poly A	4.2	24	3558	±	596
Blank	Tx/POPG/Poly A	4.2	48	4048	±	855
Blank	Tx/POPG/Poly A/NaCl	4.2	24	5888	±	108
Blank	Tx/POPG/Poly A/NaCl	4.2	48	11321	±	782

Supplementary Table 3. QuIC reaction seeded with MM1 sCJD prions. The average levels and conformational stability of PK-resistant reaction products were obtained from two independent QuIC reactions measured in triplicate with CDI and CSA. The CDI signal in TRF (cpm) is proportional to the concentration of PK-resistant PrP. The CSA GdnHCl1/2 value is a measure of conformational stability and corresponds to the midpoint of the transition from native to denatured state of PrP.

Seed	Additive	recHuPrP	QuIC Time	PK-res PrP			Conformational Stability		
		μM	hrs	CDI, TRF cpm ± S.E.M.			CSA, $GdnHCl_{1/2}$ ± S.E.M.		
MM1 sCJD	-		-	-		-	3.10	±	0.03
MM1 sCJD	GM1	4.2	48	2748	±	707		ND	
MM1 sCJD	GM1	4.2	96	8792	±	430		ND	
MM1 sCJD	NaCl	1.0	24	2473750	±	442960	3.29	±	0.04
MM1 sCJD	NaCl	1.0	48	4066500	±	109794	3.51	±	0.05
MM1 sCJD	NaCl	2.1	24	7213750	±	184544	3.22	±	0.04
MM1 sCJD	NaCl	2.1	48	6827250	±	67418	3.30	±	0.06
MM1 sCJD	NaCl	4.2	48	7056250	±	41760	3.26	±	0.03
MM1 sCJD	NaCl/GM1	4.2	48	8209	±	528		ND	
MM1 sCJD	NaCl/GM1	4.2	96	80665	±	3179		ND	
MM1 sCJD	SDS/NaCl	4.2	48	1121879	±	609456	3.28	±	0.03
MM1 sCJD	SDS/Tx/N2	4.2	24	4194	±	1366		ND	
MM1 sCJD	SDS/Tx/N2	4.2	48	1964	±	158		ND	
MM1 sCJD	SDS/Tx/NaCl	4.2	24	67331	±	4793		ND	
MM1 sCJD	SDS/Tx/NaCl	4.2	48	129463	±	2680	2.16	±	0.13
MM1 sCJD	SDS/Tx/NaCl	4.2	96	308489	±	30035	2.28	±	0.1
MM1 sCJD	Tx/GM1	4.2	24	18095	±	2985		ND	
MM1 sCJD	Tx/GM1	4.2	48	264675	±	18607	3.65	±	0.04
MM1 sCJD	Tx/Poly A/GM1	1.0	24	79228	±	2906	3.33	±	0.04
MM1 sCJD	Tx/Poly A/GM1	1.0	48	969612	±	17020	3.51	±	0.05
MM1 sCJD	Tx/Poly A/GM1	2.1	24	266724	±	13487	3.23	±	0.03
MM1 sCJD	Tx/Poly A/GM1	2.1	48	595547	±	534234	3.30	±	0.06
MM1 sCJD	Tx/Poly A/GM1	4.2	48	1441499	±	103257	3.13	±	0.03
MM1 sCJD	Tx/Poly A/GM1/NaCl	4.2	48	60281	±	20188	3.18	±	0.04
MM1 sCJD	Tx/POPG/GM1	4.2	48	13689	±	507		ND	
MM1 sCJD	Tx/POPG/GM1/NaCl	4.2	48	26134	±	1328		ND	
MM1 sCJD	Tx/POPG/Poly A	4.2	24	13075	±	713		ND	
MM1 sCJD	Tx/POPG/Poly A	4.2	48	17489	±	185		ND	
MM1 sCJD	Tx/POPG/Poly A/NaCl	4.2	24	33724	±	1925		ND	
MM1 sCJD	Tx/POPG/Poly A/NaCl	4.2	48	61802	±	5356		ND	
MM1 sCJD	Tx/POPG/Poly A/NaCl	4.2	96	127940	±	32408	3.33	±	0.04

Supplementary Table 4. QuIC reaction seeded with MM2 sCJD prions. The average levels and conformational stability of PK-resistant reaction products were obtained from two independent QuIC reactions measured in triplicate with CDI and CSA. The CDI signal in TRF (cpm) is proportional to the concentration of PK-resistant PrP. The CSA $GdnHCl_{1/2}$ value is a measure of conformational stability and corresponds to the midpoint of the transition from native to denatured state of PrP.

Seed	Additive	recHuPrP	QuIC Time	PK-res PrP			Conformational Stability		
		μM	hrs	CDI, TRF cpm ± S.E.M.			CSA, $GdnHCl_{1/2}$ ± S.E.M.		
MM2 sCJD	-	-	-	-		-	2.63	±	0.03
MM2 sCJD	GM1	4.2	48	2139	±	56		ND	
MM2 sCJD	GM1	4.2	96	12760	±	3035		ND	
MM2 sCJD	NaCl	1.0	48	6699500	±	230884	3.59	±	0.04
MM2 sCJD	NaCl/GM1	1.0	48	9841	±	790		ND	
MM2 sCJD	NaCl/GM1	2.1	96	119726	±	12963		ND	
MM2 sCJD	SDS/NaCl	2.1	48	2746500	±	762069	3.37	±	0.02
MM2 sCJD	SDS/Tx/N2	4.2	24	7586	±	1142		ND	
MM2 sCJD	SDS/Tx/N2	4.2	48	2863	±	289		ND	
MM2 sCJD	SDS/Tx/NaCl	4.2	24	98489	±	13219		ND	
MM2 sCJD	SDS/Tx/NaCl	4.2	48	250426	±	53608	2.14	±	0.1
MM2 sCJD	SDS/Tx/NaCl	4.2	96	214104	±	12166	2.34	±	0.09
MM2 sCJD	Tx/GM1	4.2	24	29979	±	1210		ND	
MM2 sCJD	Tx/GM1	4.2	48	675658	±	56088	3.68	±	0.05
MM2 sCJD	Tx/Poly A/GM1	1.0	24	340268	±	33284	3.19	±	0.05
MM2 sCJD	Tx/Poly A/GM1	1.0	48	336135	±	15054	2.93	±	0.03
MM2 sCJD	Tx/Poly A/GM1	2.1	24	621645	±	40032	3.38	±	0.03
MM2 sCJD	Tx/Poly A/GM1	2.1	48	1024191	±	32281	2.8	±	0.04
MM2 sCJD	Tx/Poly A/GM1	4.2	48	878682	±	152570	3.37	±	0.02
MM2 sCJD	Tx/Poly A/GM1/NaCl	4.2	48	2003750	±	67281	3.28	±	0.03
MM2 sCJD	Tx/POPG/GM1	4.2	48	132320	±	26132		ND	
MM2 sCJD	Tx/POPG/GM1/NaCl	4.2	48	184691	±	71604		ND	
MM2 sCJD	Tx/POPG/Poly A	1.0	24	3782000	±	148176	2.95	±	0.04
MM2 sCJD	Tx/POPG/Poly A	1.0	48	3872500	±	373604	2.93	±	0.03
MM2 sCJD	Tx/POPG/Poly A	2.1	24	5946500	±	630051	2.88	±	0.02
MM2 sCJD	Tx/POPG/Poly A	2.1	48	6900750	±	184292	2.80	±	0.04
MM2 sCJD	Tx/POPG/Poly A	4.2	24	4420750	±	259717	2.60	±	0.03
MM2 sCJD	Tx/POPG/Poly A	4.2	48	5162500	±	173996	2.80	±	0.03
MM2 sCJD	Tx/POPG/Poly A/NaCl	4.2	24	594671	±	19927	3.21	±	0.03
MM2 sCJD	Tx/POPG/Poly A/NaCl	4.2	48	4983500	±	1043916	3.47	±	0.03
