# Supplementary material for: Artificial strain of human prions created in vitro
Source: Nat Commun. 2018 Jun 4;9:2166. doi: 10.1038/s41467-018-04584-z (PMC5986862; doi:10.1038/s41467-018-04584-z)
Supplement: Supplementary file 2 — Peer Review File [file 41467_2018_4584_MOESM2_ESM.pdf]

Editorial Note: This manuscript has been previously reviewed at another journal that is not operating a transparent peer review scheme. This document only contains reviewer comments and rebuttal letters for versions considered at Nature Communications.
